# Rapid detection of tear lactoferrin for diagnosis of dry eyes by using fluorescence polarization-based aptasensor

**DOI:** 10.1038/s41598-023-42484-5

**Published:** 2023-09-13

**Authors:** Yingqi Zhang, Peng Yan, Howyn Tang, Jin Zhang

**Affiliations:** 1 Department of Chemical and Biochemical Engineering, University of Western Ontario, London, ON N6A 5B9 Canada; 2 Kensington Eye Institute, Toronto Western Hospital, Kensington Eye Institute, 600-340 College St, Toronto, ON M5T 3A9 Canada; 3 Department of Ophthalmology and Vision Science, University of Toronto, Toronto, ON M5T 3A9 Canada; 4 School of Biomedical Engineering, University of Western Ontario, London, ON N6A 5B9 Canada

**Keywords:** Diseases, Nanoscience and technology

## Abstract

Differentiating dry eye disease (DED) from allergic or viral conjunctivitis rapidly and accurately is important to ensure prompt diagnosis and treatment. Tear lactoferrin (LF), a multi-functional glycoprotein found in tears, decreases significantly in patients with DED, and has been considered as a DED diagnostic biomarker. Measuring tear LF level, however, takes time and requires the use of bulky instruments. Herein, a homogeneous carbon nanostructure-based aptasensor with high sensitivity and selectivity has been developed by applying fluorescence polarization (FP) technology. The FP of carbon dots (CDs) bioconjugated with LF aptamers (CDs-aptamer) is 21.2% higher than that of CDs, which can be further amplified (1.81 times) once interacting with graphene oxide nanosheets (GONS). In the presence of LF, GONS separates from CDs-aptamer because of the stronger binding affinity between CDs-aptamer to LF, resulting in the decrease of FP value. A linear relationship is observed between FP value and LF concentration in spiked tear samples from 0.66 to 3.32 mg/mL. The selectivity of the aptasensor has been investigated by measuring other proteins. The results indicate that the FP-based aptasensor is a cost-effective method with high sensitivity and selectivity in detection of tear LF.

## Introduction

Dysfunctional tear syndrome, usually called dry eye disease (DED), affects one in five adults in the world^1^. There are two categories of DED. Aqueous-deficiency refers to reduced tear production and hyperevaporation refers to the increased evaporation of the tear film. DED could be caused by age, certain medications, laser refractive and intraocular surgeries, and systemic diseases resulting in abnormal tear production, such as Sjogren's syndrome, rheumatoid arthritis, and collagen vascular diseases^2–4^. It is estimated that the cost for managing DED is around $55.4 billion annually in the United States^5^. One of the challenges of diagnosing and managing DED is to distinguish DED from other ocular surface diseases such as infection and allergy which have similar clinical presentations. To date, the Schirmer strip test and ocular surface staining are common methods used in clinics to diagnosis DED. In the Schirmer strip test, if the Schirmer strip is wet at less than 5 mm after direct contact with the surface of interior margin of the eye for 15 min, is diagnosed as DED^6^. Unfortunately, both the Schirmer strip test and ocular surface staining can only provide limited clinical information, preventing early diagnosis and early treatment of DED. In addition, current testing strategies are easily influenced by topical anesthesia use, and needs to consider chronic contact-lens wear and systemic medical conditions before confirming a diagnosis^7^.

Lactoferrin (LF), also known as lactotransferrin, is a multifunctional glycoprotein with a molecular weight around 80 kDa. Tear LF can bind to iron ions and minimize the inflammation which is closely associated with DED^8,9^. According to over two decades’ studies, the level of tear LF is around 0.62 ± 0.55 mg/mL in patients with DED, which is lower than that in healthy subjects, around 2.05 ± 1.12 mg/mL^10,11^. More clinical analyses have suggested that early diagnosis of DED can be benefited through monitoring the level of tear LF^6,11–13^. To date, the common methods for monitoring tear LF require experienced laboratory staff, bulky, expensive instruments, e.g., chromatography, immunoassay, electrophoresis, and additional separation and purification processes, and require several steps to determine the concentration of LF^14^. Therefore, our efforts focus on the development of a cost-effective and user-friendly sensing system to measure tear LF to achieve the rapid diagnosis of DED.

Fluorescence polarization (FP)-technology can measure the rotational motion of fluorophore-labelled molecules in homogeneous solution and it has been applied in various fields in identifying chemicals and biomolecules^15^. The degree of FP highly depends on the molecular weight of the fluorophore-labelled complex and the viscosity of the solvent^16,17^. The higher the FP value, the larger the size or weight of molecules because they normally have smaller rotational motion. The advantages of FP used in sensing systems include a) small amount of sample; b) no separation process; and c) quick detection^15,18,19^. FP-based sensing systems show superior performance in detecting proteins because most proteins have high molecular weights, resulting in lower rotation and higher FP value^20^.

**Figure 1:**
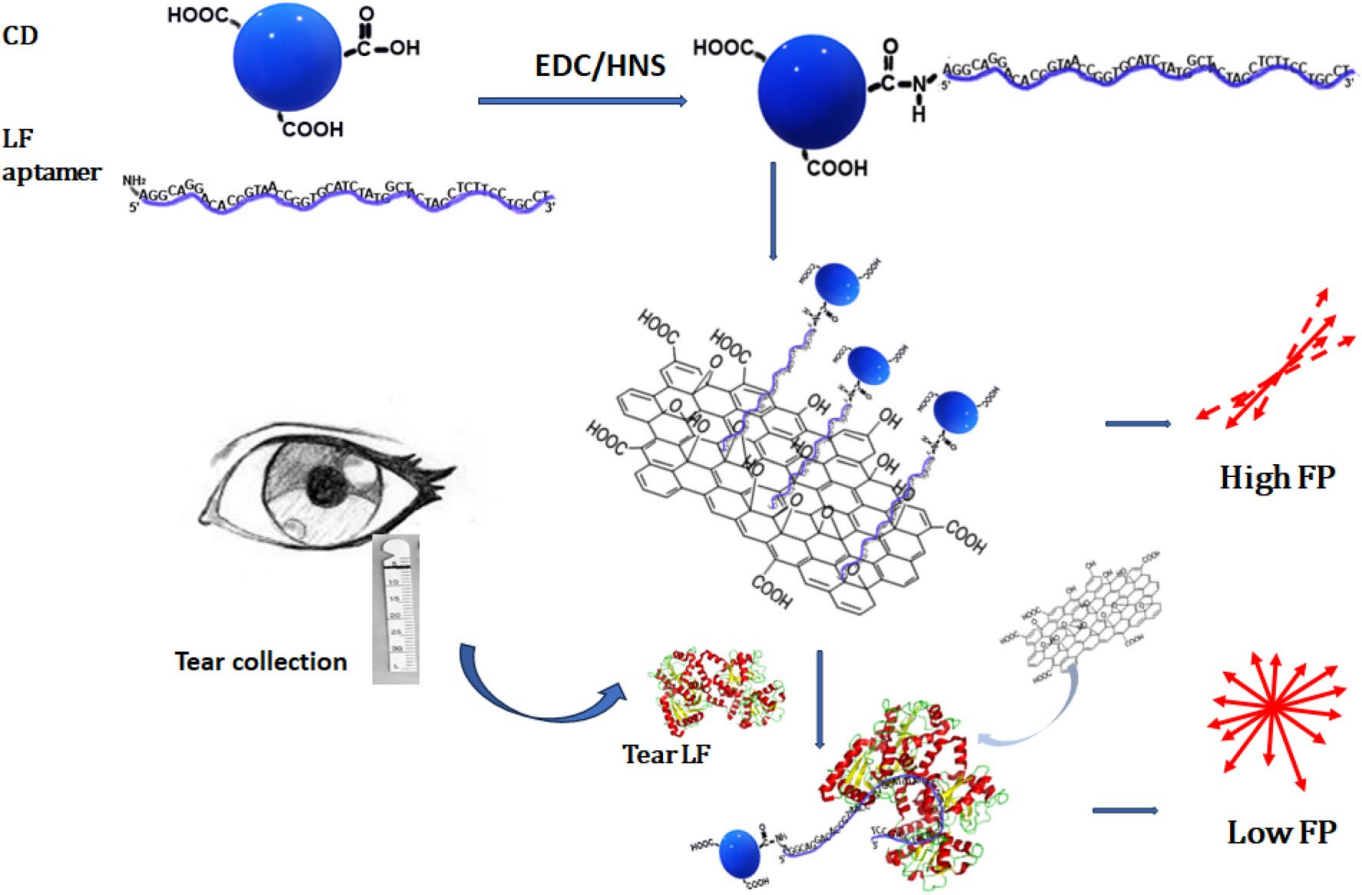
Scheme of FP-based aptasensor for the detection of lactoferrin.

Nanomaterials employed in FP-based biosensing system not only act as FP signal generators, e.g., quantum dots^21^ but also work as amplifiers to enhance FP signals, including gold nanoparticles, silver nanoparticles, and graphene oxide^22–24^. Carbon dots (CDs) are emerging carbon-based nanostructures which show special tunable luminescence and good biocompatibility^25^. CDs have demonstrated versatile capabilities in binding/bioconjugating with different chemicals and biomolecules, which allow them to act as fluorescent probes to detect various biomolecules^26,27^. While very few CDs have been used in FP-based sensing system, herein, a FP-based sensing system made of LF aptamer-conjugated CDs has been developed for quickly detecting lactoferrin from spiked tear samples (as shown in Fig. 1). On the other hand, aptamers normally show strong and specific binding affinity to target molecules, including nucleic acids and proteins^28^. In addition, aptamers can be bioconjugated onto the surface of nanomaterials with enhanced chemical and physical properties to form portable sensing systems^29,30^. A protein microarray microfluidic chip for screening aptamers (PMM-SELEX) was recently developed by Danke Xu’s group by using LF from bovine milk as the target protein^31^. The dissociation constants (K_d_) of the synthetic nucleic acid aptamers for binding to LF are in the range of 5.48 ± 1.79 to 0.63 ± 0.06 nM. The same group further reported using silver nanoparticles enhanced FP to detect LF in milk powder^32^. The results indicate that the aptamer candidates with stem-loop structure show high affinity with LF. On the other hand, graphene oxide nanosheets (GONSs) have been reported to act as FP amplifiers^32^. As per previous study, single strand DNA can be absorbed on the surface of GONSs through the π-π* stacking interaction of the nucleobases^33–35^. Thus, a solution-based aptasensor made of carbon-based nanostructures is developed to detect spiked tear LF. GONSs acting as the FP amplifier coupled with LF aptamer-conjugated CDs via π-π* interaction. In the presence of LF, GONSs will be apart from LF aptamer-conjugated CDs which leads to the decrease of FP value because the affinity between LF aptamer and lactoferrin is stronger than the π-π* interactions between aptamer and GONSs. This study demonstrates a cost-effective detection method to quickly and quantitatively measure tear LF with high sensitivity and selectivity.

## Materials and methods

### Materials and reagents

Citric acid (251,275), urea (U5128), PBS Tablet (524,650), N-(3-Dimethylaminopropyl)-N′-ethylcarbodiimide hydrochloride (03450), N-Hydroxysuccinimide (130,672), lactoferrin from bovine colostrum (L4765), glucose oxidase from aspergillus niger (G7141), concanavalin A-peroxidase from *Canavalia ensiformis* (Jack bean) (L6397), β-casein from bovine milk (C6905), lysozyme human (L1667), bovine serum albumin (B4287), potassium permanganate (223,468) and hydrochloric acid (320,331) were purchased from Sigma-Aldrich. Graphite flake (43,209) used for fabricating GONSs was purchased from Alfa Aesar. Sulphuric acid and magnesium chloride (105,672) were purchased from Caledon Laboratory Chemicals. The lactoferrin aptamer was purchased from Integrated DNA Technologies^31,36^.

### Synthesis of carbon dots (CDs)

The water-soluble fluorescent carbon dots were synthesized through one-step approach by using the microwave-assisted process based on previous reports^37^. Citric acid and urea with a ratio of 1:1 dissolved in distilled water, which was then heated by a Panasonic 750 W microwave for 5 min. The product was heated in a vacuum oven for 1 h at 60 °C and centrifuged for 20 min at 3000 rpm. The final product, in powder, was kept at 4 °C for future uses, after 2 h dialysis against distilled water followed by the lyophilization process. The photoluminescence of CDs has been studied and can be found in Fig. S1.

### Bioconjugation of LF aptamers on CDs

The fluorescent probe consists of two parts: CDs are used for generating fluorescent signal and LF aptamers are considered as the bioreceptor to identify lactoferrin. LF aptamers with a sequence of 5’-AGG CAG GAC ACC GTA ACC GGT GCA TCT ATG GCT ACT AGC TCT TCC TGC CT-3’ purchased from Integrated DNA Technologies was used to modify the surface of CDs, which was modified with amino on the 5’ end. As per a previous study, the K_d_ of this LF aptamer is 4.97 ± 0.46 nM^36^. Furthermore, this LF aptamer has a lower ΔG, − 5.82 kcal mol^−1^, which indicates the good stability of this LF aptamer in PBS buffer^31^. Carbodiimide conjugation by using N’-(3-dimethylaminopropyl)-N-ethylcarbodiimide (EDC) and N-hydroxysuccinimide (NHS) had been investigated to bioconjugate biomolecules on CDs^37–39^. To conjugate LF aptamer onto CDs, 20 mM EDC and 20 mM NHS were introduced into the CDs solution with concentration of (0.1 mg/mL). 1 mL mixture of CD, EDC, NHS was then subjugated to shaking in the dark for 20 min to activate the carboxylic groups on the surface of CDs. Then, 100μL LF aptamers with concentration of 10 μM modified with amino on the 5’ end was dropped into the previous solution and then put in the dark at 4 °C with stirring at 400 rpm overnight to obtain CDs-aptamer. The final product was stored at 4 °C for future use after 2 h dialysis against distilled water. To optimize the ratio of CDs to aptamer, electrophoresis had been employed (Fig. S2). The measurement had been repeated with three times.

### Synthesis of graphene oxide nanosheets (GONSs) acting as the FP amplifier

Graphene oxide nanosheets (GONSs) were obtained by following a modified version of Hummer’s Method^40^. 1 g graphite flake was added into 50 mL 98% sulfuric acid (H_2_SO_4_) with 5 min stirring in water bath at 0 °C. 3 g potassium permanganate was added followed by the stirring-sonication process (stirred for 25 min at a temperature under 10 °C and then sonication for 5 min in ultrasonic bath). After the stirring-sonication process was repeated 12 times, 200 mL of distilled water was added, followed by sonication for 2 h. Finally, hydrochloric acid solution and distilled water was utilized to wash GONSs with centrifuged at 8000 rpm for 30 min until pH 6. GONSs powder was obtained by the lyophilization process.

### Materials characterization

Carbon-based nanostructures including CDs and GONSs were characterized by transmission electron microscopy (TEM; Phillips CM-10 at 80 kV). The diameter of the CDs from the TEM images was analyzed with ImageJ software (National Institutes of Health, USA). Photoluminescence of CDs was studied by using a Spectrofluorometer (QuantaMaster™ 40, Photon Technology International Inc.). The surface modification and bioconjugation of CDs were investigated by using Fourier Transform Infrared (FTIR, Bruker Vector 22), in the range of 600–4500 cm^−1^ with a resolution of 4 cm^-1^ and 64 scans. Hydrodynamic diameter and zeta potential of CDs and CDs-aptamer were measured by Mastersizer (Malvern Instruments Ltd). In addition, agarose gel electrophoresis was carried out to verify the bioconjugation of aptamer to CDs. The gel was visualized under a UV Transilluminator with excitation at 365 nm (Fig. S2). It is noted that the emission of CDs at excitation of 365 nm is 450 nm as shown in Fig. S1.

### Sensing performance on tear samples

The FP values were measured by using a PicoQuant FluoTime 200 fluorescence lifetime spectrometer with the excitation wavelength (λ_ex_) = 405 nm. The fluorescence emitted at λ_em_ = 535 nm at the horizontal (${\text{I}}_{//}$) and vertical (${\text{I}}_{ \bot }$) directions, respectively, were obtained. The value of FP is calculated as per Eq. (1)^41^.

1 $$FP = \frac{{I_{ \bot } - I_{//} }}{{I_{ \bot } + I_{//} }}$$

As per previous studies, the fluorescence resonance energy quenching occurs when the CDs-aptamer are absorbed on the surface of GONSs, the optimal ratio of CDs-aptamer to GONSs have been studied to remain suitable photoluminescence (Fig. S3). In the measurement of FP of the carbon nanostructured sensing system, 5 uL CDs-aptamer (0.5 mg/mL) were incubated with 45 uL GONSs (0.055 mg/mL) in phosphate-buffered saline (PBS) buffer at pH = 6.8. It is noted that the concentration of CDs remained at 0.05 mg/mL in all FP measurements. Different concentrations of lactoferrin (LF) in PBS buffer at pH = 6.8 from 2 to 10 μg/mL were introduced in the sensing system with 30 min incubation in dark at 37 °C (Fig. S4 shows the effect of incubation time on sensing performance). Figure S5 shows the standard curves of FP value as the function of the concentration of LF in aqueous media. The limit of detection (LOD) of this sensing system for detecting LF in aqueous media is 1.397 μg/mL.

Human tear samples were collected by using the Schirmer Strip and then extracted (informed consent was obtained from all subjects and/or their legal guardian(s)). Samples were stored at − 80 °C for future use. Extracted tear sample from the Schirmer Strip was diluted 250 times in sensing measurement. Meanwhile, the Human LTF/Lactoferrin ELISA Kit purchased from Invitrogen was used to measure spiked tear samples to compare with the results from FP-based LF aptasensor in determining the concentration of lactoferrin in spiked human tear. The ELISA test was taken with triple measurements, and each measurement has been taken duplicated (that is, six readings can be obtained for each sample). The control (LF in PBS buffer) and blank samples have been used in the measurement. Each sample was diluted and measured six times by LF ELISA test and FP_based LF aptasensor, respectively. The mean and standard deviation (SD) were calculated.

In addition, the selectivity of the designed FP-based LF aptasensor has been evaluated by measuring different proteins in in 10 μg/mL, including human lysozyme, glucose oxidase from *Aspergillus niger*, concanavalin A-peroxidase from *Canavalia ensiformis*, β-casein from bovine milk, and LF from bovine colostrum. The normalized FP value is calculated by using Eq. (2).

2 $$Normalized\, FP=\frac{{FP}_{protein} -{FP}_{PBS} }{{FP}_{PBS} } 100\%$$

where *FP*_*protein*_ is the FP value when the protein sample is introduced in the sensing assay, and *FP*_*PBS*_ is the FP value without introducing protein sample.

### Ethics approval

Ethics approval is obtained from the University of Toronto Research Ethics Board, in accordance with the Canadian Council on human involved research guidelines. Informed consent was obtained from all subjects and/or their legal guardian(s).

## Results and discussion

### Development of CDs-aptamer

**Figure 2:**
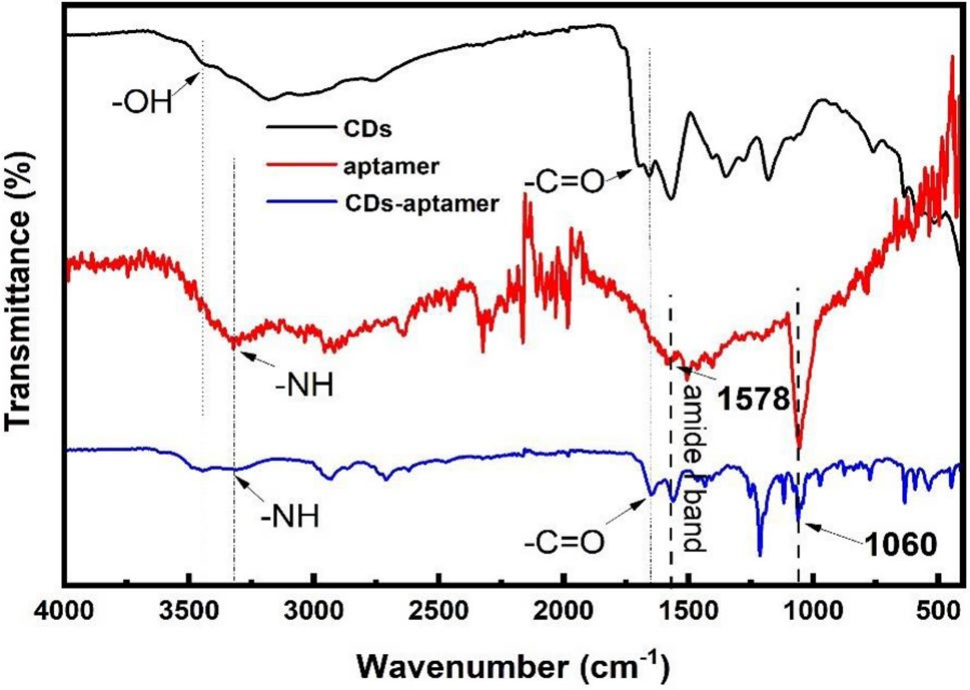
FTIR spectra of CDs, aptamer, and CDs-aptamer.

Figure 2 is the FTIR spectra of free CDs, aptamer, and CDs-aptamer. To free CDs, the band at 3440 cm^−1^ is attributed to the C–OH stretching vibration, and the peak at 1697 cm^−1^ is related to C=O stretching vibration. The results demonstrate that carboxylic groups are observed on the surface of CDs^42,43^. The wide peak around 3320 cm^−1^ and the peak at 1578 cm^−1^ displayed in the FTIR spectrum of aptamer are attributed to N–H bending vibration, which are caused by the amino group modified on the 5’ end of aptamer. To CDs-aptamer, the peaks observed around 3320 cm^−1^ and 1647 cm^−1^ can be attributed to the N–H stretching and C=O stretching, respectively, which stem from the formation of –CO–NH– from the reaction between carboxyl on the surface of CDs and amino functional group modified on the 5’end of LF aptamer^44^. Meanwhile, the peak around 1060 cm^−1^ can be observed in both FTIR spectra of aptamer and CDs-aptamer, which is related to the phosphate group. The result shows that the single-stranded aptamer remains after the bioconjugation onto CDs.

To further understand the bioconjugation of LF aptamer on CDs, free CDs, and CDs-aptamer with different ratios of CDs to LF aptamer have been studied by using the agarose gel electrophoresis. A full-length gel can be found in the supplementary information file as shown in Fig. S2. The movement of CDs-aptamer is similar with the free CDs when the ratio of mass concentration (mg/mL) of aptamer to CDs increases from 0.4 to 1.6. The movement of CDs-aptamer is slower as compared with that of free CDs when the ratio of aptamer to CDs reaches 3.6. The slowest movement in gel to CDs-aptamer is observed when the concentration ratio of CDs to LF is around 1:6.4. Additional aptamer beyond this ratio added in the system could not increase the amount of aptamer bioconjugated on CDs. Thus, the sensing system contains CDs-aptamer with the ratio (mg/mL) of CDs to LF around 1:6.4.

### Characterization of carbon-based nanostructures

**Figure 3:**
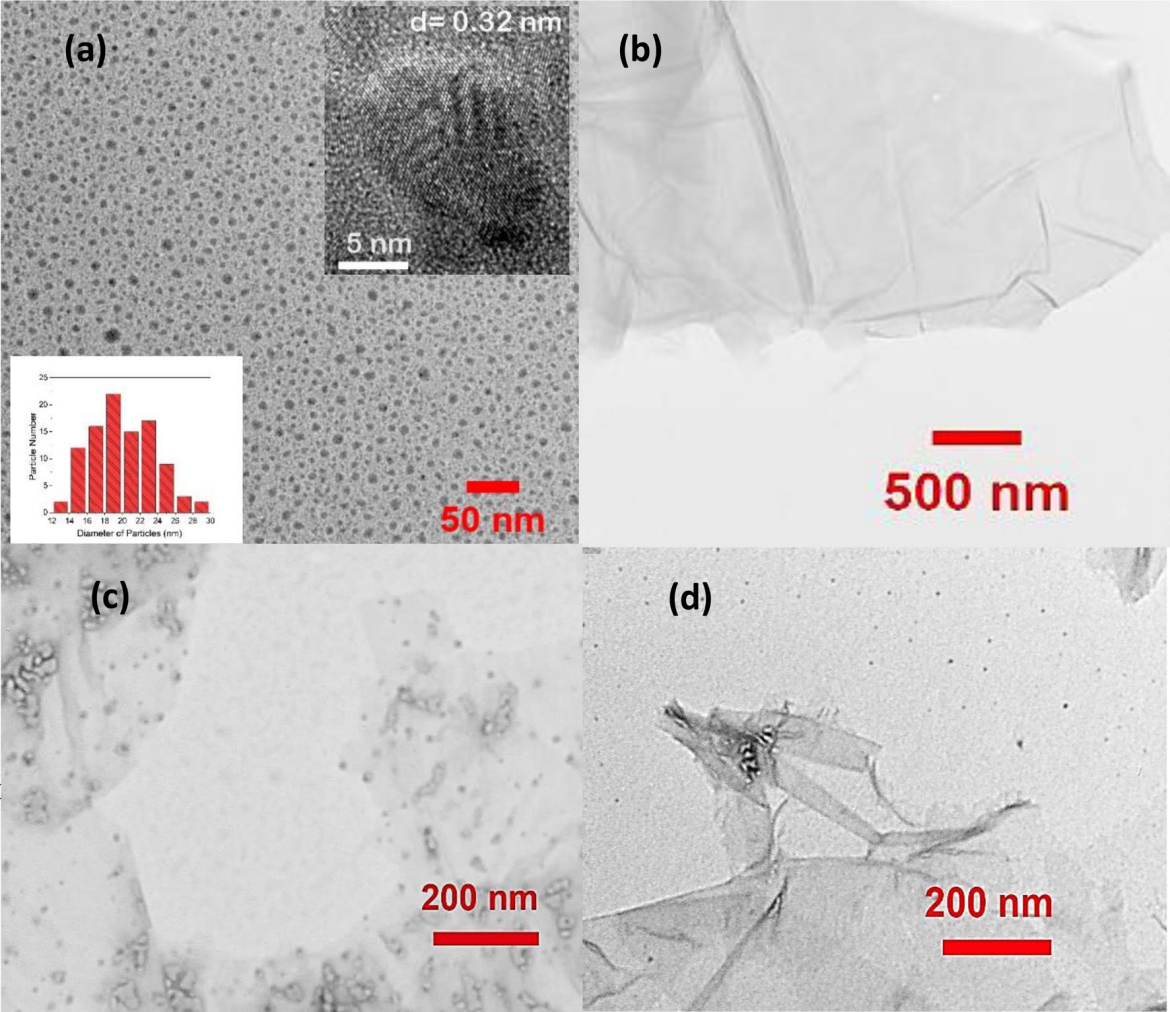
TEM micrographs (**a**) carbon dots (CDs); the small insets are the size distribution and the HRTEM micrograph of CDs (**b**) GONS; (**c**) CDs-aptamer-GONSs; (**d**) CDs mixed with GONSs.

Figure 3 are the TEM micrographs of carbon-based nanostructures. The average particle size of CDs is around 20.3 ± 3 nm as shown in Fig. 3a. It is similar with the result of the average particle size of CDs, 20.7 nm, measured by using dynamic light scattering (DLS) (see the supplementary file, Fig. S6). The small inset at the right corner of Fig. 3a is the high resolution TEM (HRTEM) micrograph of CDs. The interplanar distance (*d*) is around 0.32 nm, corresponding to (002) lattice spacing of graphite carbon^45^. Figure 3b shows the TEM micrograph of GONS which displays slight folds and wrinkles on the surface of single layer GONSs. In addition, the typical UV absorbance peak of thin layer of GONSs can be observed at 230 nm as shown in Fig. S7 (see the supplementary file)^46^. The weak shoulder peak at 300 nm indicates that the GONSs is a single layer which has been verified by TEM (Fig. 3b)^43^. Figure 3c is the TEM micrograph of CDs-aptamer absorbed on GONSs. The feature of single strand DNA (LF aptamer, 50 bp) attached on the CDs can be observed. According to previous study^33–35^, π-π interaction is the main force between single strand DNA and GONSs as DNA bases contain aromatic and hydrophobic rings. The particle size of CDs before and after interacting with GONSs remains similar. No significant aggregation is observed which indicates that the surface of CDs has been covered by aptamer via the bioconjugation of aptamer with maximum ratio of aptamer to CDs. In addition, the CDs without aptamer-conjugation mixed with GONSs. Due to the repulsive interaction among nanostructures and fine particles, CDs are separated from GONSs as shown in Fig. 3d, very few CDs can be observed on the surface of GONSs. This result also indicates that GONSs acting as the FP amplifier, can allow CDs absorbing/assembling on the small area of GONSs to increase the overall mass of CDs resulting in high FP value.

### Fluorescence Polarization (FP) of carbon nanostructure-based systems

**Figure 4:**
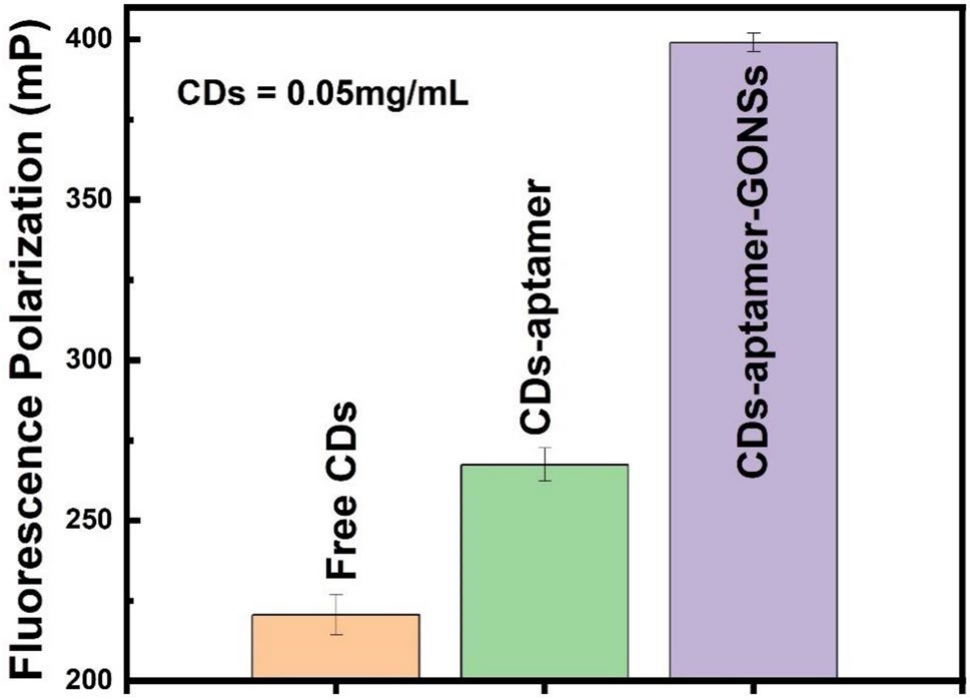
FP value of free CDs, CDs-aptamer and CDs-aptamer-GONSs.

The FP value of carbon nanostructure-based systems, i.e., free CDs, CDs-aptamer, and CDs-aptamer-GONSs, highly depends on their size and molecular weight. As per Eq. (1), the larger the size/molecular weight of the system, the higher the FP value. The FP value of CDs with the concentration of 0.05 mg/mL has been measured. Figure 4 shows the FP value of the fluorescent CDs is 220 ± 5 mP, which can be increased to over 21.2% after conjugating with aptamers, and the FP value can be further amplified to over 49.2% after the fluorescent probe (CDs-aptamer) is bonded on GONSs. The results indicate the successful bioconjugation of aptamer onto CDs which can be attached on the surface of GONSs via the π-π interaction between aptamers and GONSs; and GONSs can act as the amplifier of FP to CDs.

### Performance of the biosensing system in measuring tear LF

**Figure 5:**
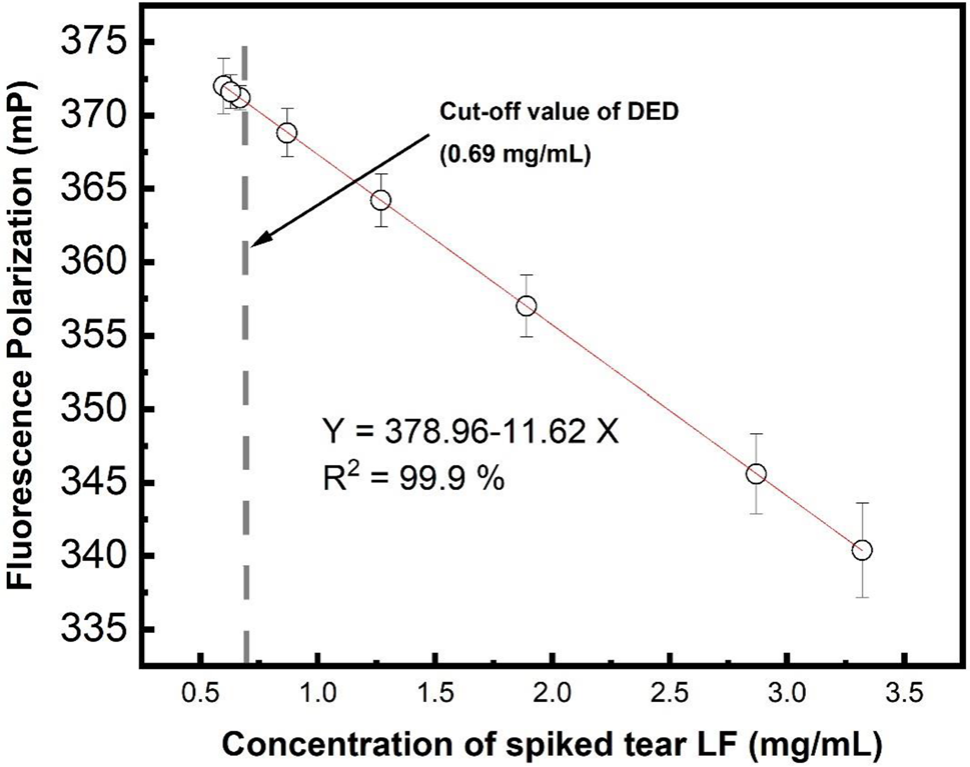
FP as a function of LF from spiked tear samples.

Figure 5 shows that the FP value increases when the concentration of spiked tear LF decreases from 1.89 to 0.6 mg/mL. A linear relationship between the FP level and the concentration of tear LF from spiked tear samples, which can be expressed with the equation of Y = 378.96–11.62 X, where Y is the FP value, X is the concentration of the spiked tear LF. It is noted that the cut-off value of DED is 0.69 mg/mL, which has been reported by using different methods to detect tear LF as a biomarker for diagnosis of DED^10,11,47^. Meanwhile, the concentration of human tear LF and spiked tear LF were measured (Table 1) by using both the FP-based aptasensor and the Human LTF/Lactoferrin ELISA Kit. The ELISA results indicated that the tear LF collected from human tear sample is 3.742 ± 1.276 mg/mL. The concentration of LF in the same human tear sample is 3.32 ± 0.27 mg/mL which was measured by the developed FP-based LF aptasensor. Meanwhile, the samples of spiked tear LF was measured by both the ELISA and the designed FP-based LF aptasensor. The trend of the measured concentration of spiked LF is the same for both sensing systems. It is noted that the discrepancy in the concentration of spiked tear LF measured by Human LTF/Lactoferrin ELISA Kit and our FP-based LF aptasensor, respectively, is smaller when the concentration of LF is decreased. The standard deviation of LF concentration measured by the FP-based LF aptasensor is about half of that measured by ELISA. The results also indicate that FP-based LF aptasensor has a lower standard deviation (SD) in measuring lower concentrations of LF as compared to ELISA measurement. It is noted that LF ELISA kit uses the antibody-LF interaction, while our solution-based sensing system utilizes the interaction of LF aptamer and LF. Table S1 shows the comparison between antibody and DNA-based aptamer. In addition, the selectivity of the FP sensing system has been evaluated. Different proteins, lysozyme, oxidase, peroxidase, and β-casein with the same concentration of LF have been measured by the FP sensing system.

**Table 1:** Concentration of tear LF determined by FP-based aptasensor as compared with the result of ELISA.

	ELISA kit (mean ± SD) (mg/mL)	FP-based aptasensor (mean ± SD) (mg/mL)
Sample 1 Human tear sample	3.93 ± 0.50	3.32 ± 0.27
Sample 2 Spiked tear sample	1.34 ± 0.24	1.27 ± 0.15
Sample 3 Spiked tear sample	0.97 ± 0.26	0.87 ± 0.14
Sample 4 Spiked tear sample	0.69 ± 0.18	0.66 ± 0.09

**Figure 6:**
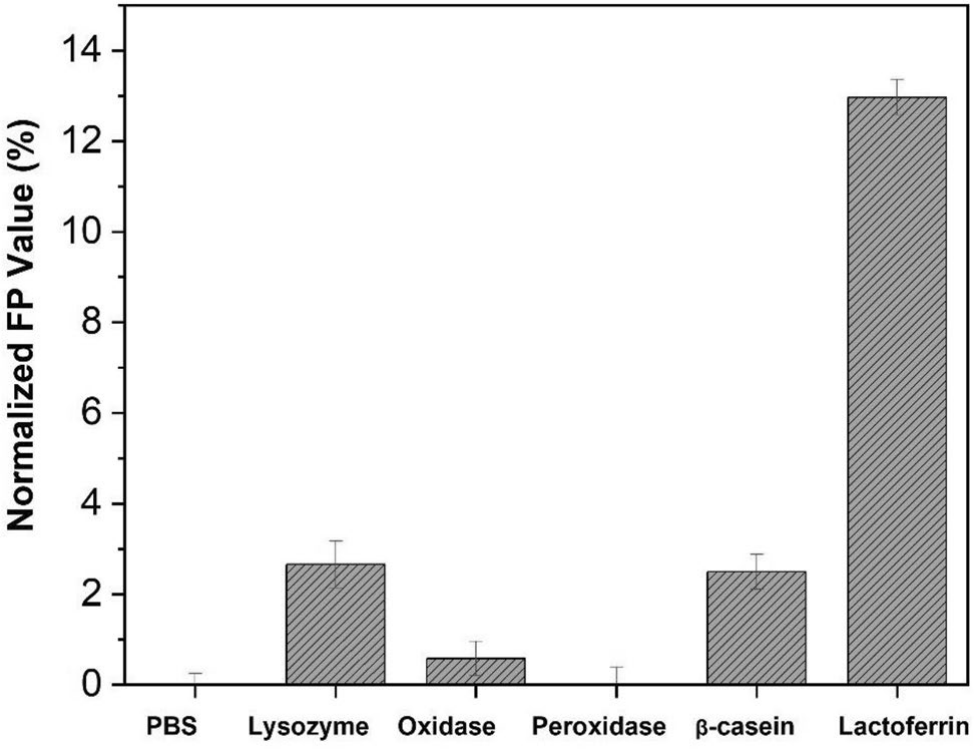
Investigation of the selectivity of the FP-based LF aptasensor. Proteins include: (1) Lysozyme: human lysozyme; (2) Oxidase: glucose oxidase from *Aspergillus niger*; (3) Peroxidase: concanavalin A-peroxidase from *Canavalia ensiformis*; (4) β-Casein: β-casein from bovine milk; (5) Lactoferrin: lactoferrin from bovine colostrum.

Figure 6 shows the normalized FP value when the proteins have been introduced in the sensing assay, respectively. The normalized FP value does not change when glucose oxidase and peroxidase are introduced in the sensing system, respectively. Other proteins added in the sensing system only led to a slight decrease of FP, less than 6%; whereas the change of FP value is over 12% when the same concentration of LF is introduced in the sensing assay. The result indicates that the FP sensing system has a high selectivity in detecting spiked tear LF.

It is noted that GONS can act as a FP amplifier in this carbon nanostructures-based aptasensor because numerous CDs-aptamer can be absorbed/assembled on a very small area of the surface of GONSs via π-π interaction between aptamer and GONSs. As shown in Fig. 3b, hundreds of CDs-aptamer can be absorbed on the surface of GONS (500 nm × 200 nm). This phenomenon can cause a larger overall mass of the source of FP, i.e., CDs’ mass in a small dimension, resulting in a higher FP value of the system. Since the binding between GONS and the aptamer is weaker than the binding between the aptamer and LF, CDs-aptamer will preferentially bind to LF when it is present, and can be suspended in aqueous solution individually. Due to the three dimensional chemical structure of LF and the limited number of aptamers conjugated onto CDs, only a number of LF can be bound to single CD, which results in the decrease of the mass of the source of FP as compared with that of CDs-aptamer absorbed on the surface of GONSs. Therefore, the FP value decreases when the concentration of LF increases while CD-aptamer is apart from GONSs. This study first demonstrates that GONSs acting as the FP amplifier in this carbon nanostructured aptasensor can lead to a great difference in FP value between CDs-aptamer-GONS and CDs-aptamer, and can detect a wide range of LF with high sensitivity.

Compared to other detection methods, there are several benefits to this method such as the high FP amplification obtained by using GONS, low cost, ease of operation, and rapid response time. It took 30 min to receive the detection result by using the FP-based aptasensor made of carbon nanostructures. Whereas it took over 2 h to receive detection result by using LF ELISA kits which is relatively expensive. In addition, the limit of detection (LOD) of this sensing system is 1.397 μg/mL (see section of S4, Fig. S5). On the other hand, since CDs-aptamer is adsorbed onto GONSs, there may be nonspecific desorption, more studies are needed. In addition, a suitable aptamer is vital to achieve high sensitivity and selectivity. As such, studies on integrating other aptamers onto carbon nanostructures are required.

## Conclusions

In summary, a cost-effective and user-friendly FP sensing system made of carbon-based nanostructures has been developed to quickly detect spiked tear LF. CDs with emission wavelength at 535 nm under the excitation wavelength at 405 nm can be used to generate the FP signal. In addition, the FP amplifier of CDs is made of GONSs which allow numerous CDs-aptamer absorbing/assembling on the small area of the surface of GONSs through π-π interactions between aptamer and GONSs. The FP value of CDs-aptamer-GONS is 1.81 times the FP value of CDs. A linear relationship between FP and the concentration of tear LF from the spiked tear samples from 0.6 to 3.32 mg/mL. The sensing system has a limit of detection of around 1.397 μg/mL. No separation and purification processes are required in the detection. The performance of FP-based aptasensor used to measure LF from spiked tear samples has been evaluated through the comparison of the results obtained by using human lactoferrin ELISA kit. The results show that the FP-based aptasensor made of carbon nanostructures can provide an alternative solution to rapidly measure tear LF with higher accuracy. The FP-based aptasensor made of carbon nanostructures has potential in the prompt diagnosis of DED with high sensitivity and selectivity as well to allow early treatment intervention.

### Supplementary Information

Supplementary Information.

## Data Availability

Data will be made available on request. In order to receive data from this study, please contact Jin Zhang: jzhang@eng.uwo.ca.
