# Supplementary material for: Rapid detection of tear lactoferrin for diagnosis of dry eyes by using fluorescence polarization-based aptasensor
Source: Sci Rep. 2023 Sep 13;13:15179. doi: 10.1038/s41598-023-42484-5 (PMC10499909; doi:10.1038/s41598-023-42484-5)
Supplement: Supplementary file 1 — Supplementary Information. [file 41598_2023_42484_MOESM1_ESM.pdf]

Supplementary materials

Rapid Detection of Tear Lactoferrin for Diagnosis of Dry Eyes by Using Fluorescence Polarization-based Aptasensor

Yingqi Zhang[1], Peng Yan[2,3], Howyn Tang[4], Jin Zhang[1,4]*

[1] Department of Chemical and Biochemical Engineering, University of Western Ontario, London, Ontario, Canada N6A 5B9

[2] Kensington Eye Institute, Toronto Western Hospital, Kensington Eye Institute, 600-340 College St, Toronto ON M5T 3A9

[3] Department of Ophthalmology and Vision Science. University of Toronto

[4] School of Biomedical Engineering, University of Western Ontario, London, Ontario, Canada N6A 5B9

*** Corresponding Author**

Jin Zhang, Ph.D.

Dept. of Chemical & Biochemical Engineering

University of Western Ontario

London ON. Canada N6A 5B9

Tel: 519 661 2111 ext. 88322

Email: jzhang@eng.uwo.ca

S1. Photoluminescence of CDs

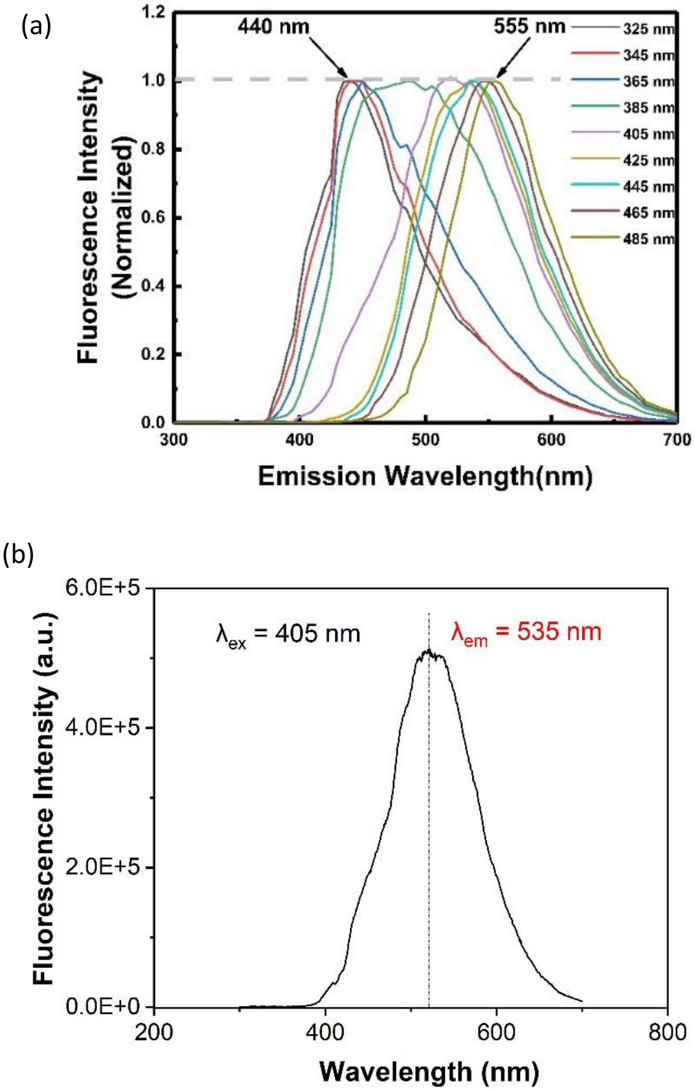

Fig. S1 (a). Photoluminescence (PL) of CDs under different excitation wavelengths. **(b)** Photoluminescence (PL) of CDs when λ_{ex} = 405 nm

Fig 1a shows that emission of CDs can be tuned by changing the excitation wavelengths from 325 nm to 485 nm.

Fig. 1b indicates, when λ_{ex} = 405 nm, the maximum intensity (I_{max}) is centering at 535 nm.

S2. Optimal ratios of carbon nanostructures and aptamer used in the homogeneous aptasensor

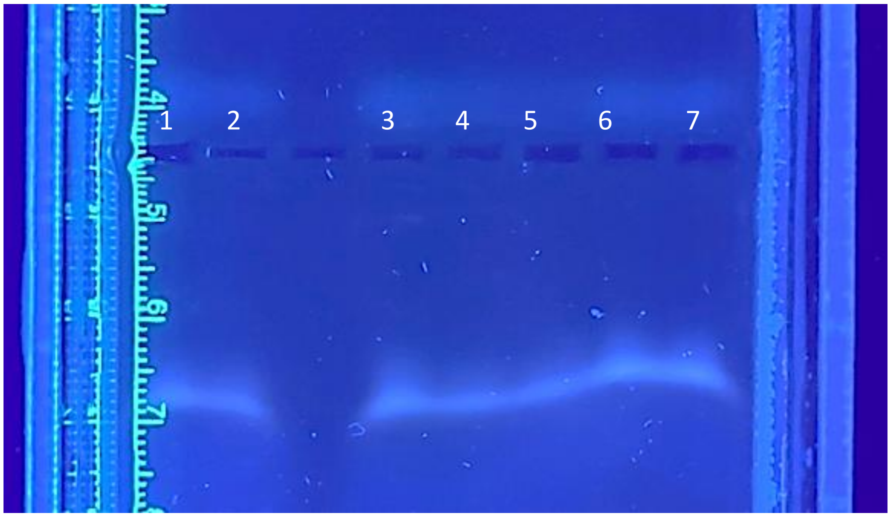

Fig. S2. Agarose gel electrophoresis to verify the bioconjugation of aptamer to CDs. The original photo of the gel after running the electrophoresis which was used to study the bioconjugation of aptamer to CDs. The agarose gel electrophoresis was carried with samples of free CDs and 0.02 mg CDs conjugated with different volumes of 10 μM aptamers. Here, Sample 1 & 2: free CDs; Sample 3: CDs-aptamer (CDs: aptamer = 1:0.4); Sample 4: CDs-aptamer (CDs: aptamer = 1:1.6); Sample 5: CDs-aptamer (CDs: aptamer = 1:3.6); Sample 6: CDs-aptamer (CDs: aptamer = 1:6.4); Sample 7: CDs-aptamer (CDs: aptamer = 1:10.0).

Sample 6 CDs: aptamer = 1:6.4) is used in following study.

.

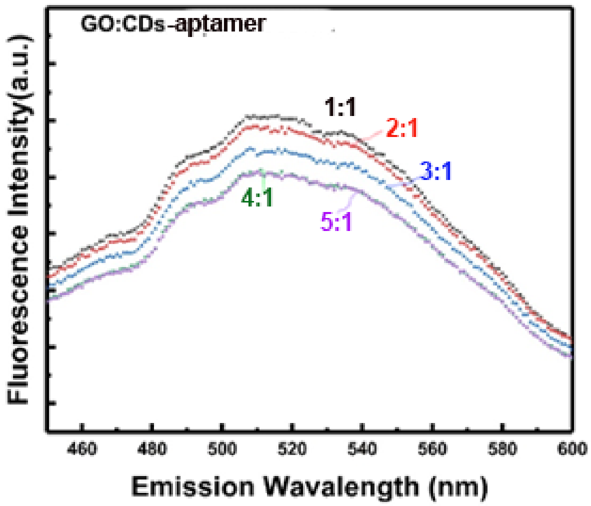

Fig. S3. Photoluminescence (PL) of CDs-aptamer-GONS system; the concentration ratios of CDs-aptamer to GO are from 1:1 to 1:5, the excitation wavelength at λ_{ex} = 405 nm. Due to fluorescence resonance energy quenching, the concentration ratio of CDs-aptamer to GO is 1:1 in this study.

S3. To determine the incubation time when introducing LF in the homogeneous aptasensor

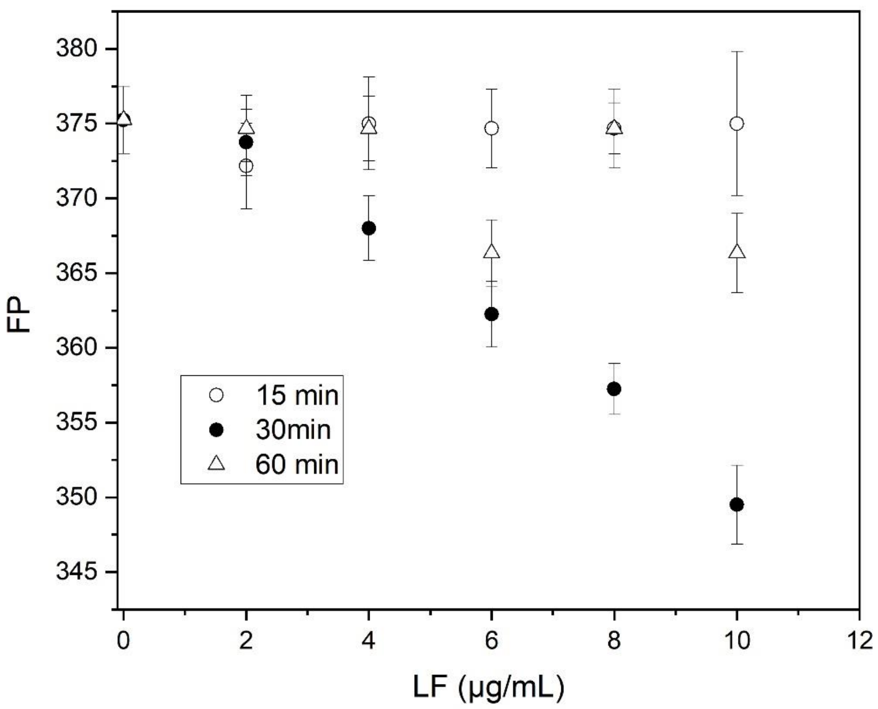

Fig. S4. FP as a function of the concentration of LF when incubation time (*t*) = 15 min, 30 min, 60 min. *t* = 30 min is chosen in studying the sensing performance.

S4. Standard curve of FP vs. the concentration of LF in aqueous media

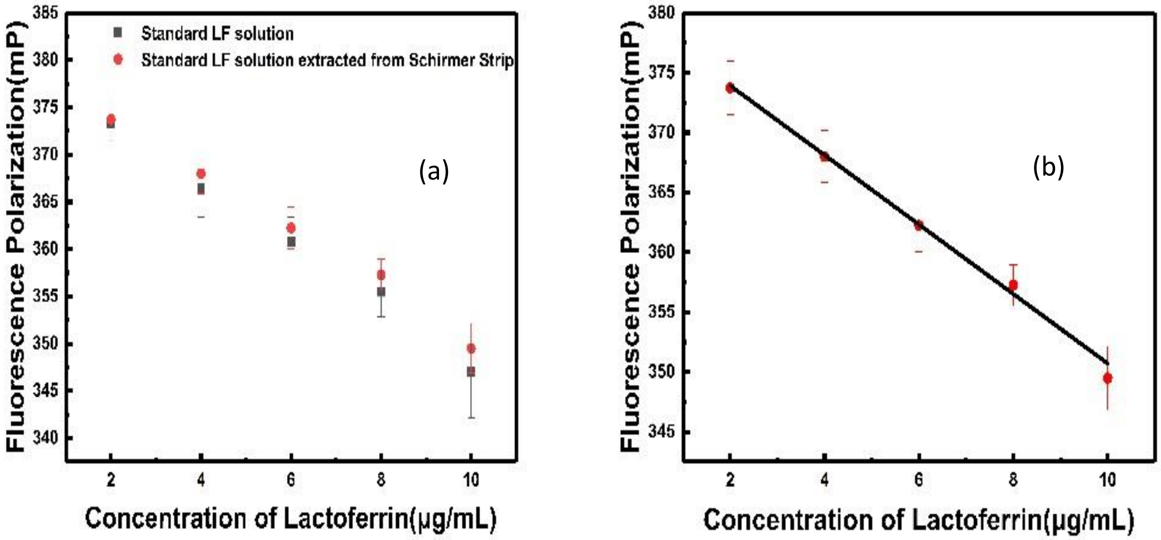

Fig. S5. (a) FP signal as a function of lactoferrin (LF) in aqueous media. (b) A linear relationship between FP value and the concentrations of aqueous LF solution extracted from Schirmer Strip.

Standard LF solution was prepared with different concentrations from 2 μg/mL to 10 μg/mL (black dots in Figure S5a). In addition, the standard LF solution dropped on and then extracted from the Schirmer Strip were also measured by FP to confirm that LF in tear samples can be collected and extracted by using the Schirmer Strip (red dots in Figure S5a). A linear relationship (Figure S5b), Y=379-2.9X where Y is FP value and X is concentration of LF, is obtained to indicated FP value and the concentration of lactoferrin. According to IUPAC, the limit-of-detection (LOD) is a measured quantity value as shown Equation 1[1];

The limit of detection (LOD) = $k\frac{\sigma_B}{b}$ (1)

where σ_B is the standard deviation of FP value of the aptasensor without introducing LF and b is the slope of the standard curve, FP signal as a function of LF concentration.

In this study, LOD of this sensing system is 1.397 μg/mL.

S5. Analysis of CDs by using Dynamic Light Scattering (DLS)

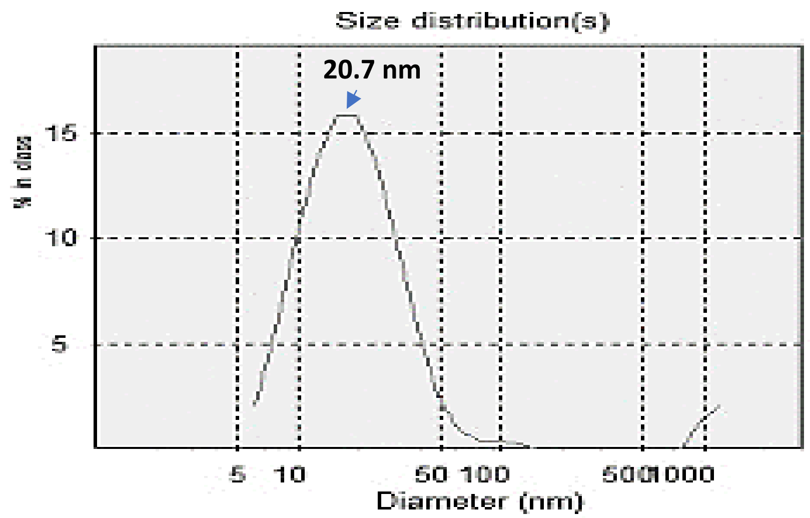

Fig. S6. Size analysis of CDs by using dynamic light scattering (DLS).

S6. UV absorbance of GONSs

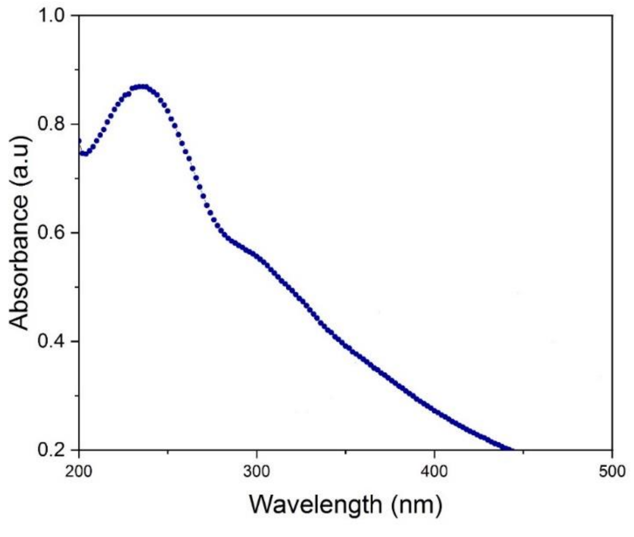

Fig. S7. UV-vis absorbance of GONSs.

S6. The cost and operation time of LF ELISA kit vs. FP-based aptasensor

S7. Comparison between aptamer-based assay and antibody testing

Table S1. Bioreceptors: DNA-based aptamer vs. antibody.

	DNA-based Aptamer	Antibody
Thermal/chemical stability	Aptamers are stable under different conditions[2]	Antibodies are susceptible to high temperatures and pH[3]
Chemical modification to bound to sensor transducer	Easy as it can be modified at both the 5' and 3'end.	Relatively hard because of the large Y-shaped glycoprotein.
Reproducibility	Greater batch-to-batch consistency[2]	Batch-to-batch consistency is a problem[2-3]
Producing	A chemically synthesized process[2]	A time-consuming biological process[2]
Price	Cheap.<br>< CAD $100 (100 µl)<br>www.idtdna.com	Expensive.<br>US $540~600 (100 µl)<br>www.Abcam.com

References

[1] Desimoni E, Brunetti B (2015) About Estimating the Limit of Detection by the Signal to Noise Approach. Pharm Anal Acta 6: 355.

[2] Bauer, M.; Strom, M.; Hammond, D. S.; Shigdar, S., Anything you can do, I can do better: Can aptamers replace antibodies in clinical diagnostic applications? Molecules 2019, 24 (23), 4377

[3] Le Basle, Y.; Chennell, P.; Tokhadze, N.; Astier, A.; Sautou, V., Physicochemical stability of monoclonal antibodies: a review. Journal of Pharmaceutical Sciences 2020, 109 (1), 169-190.
